# Enhanced orbital adipogenesis in a mouse model of T-cell-mediated autoimmunity, zymosan A-treated SKG mice: Implications for Graves’ ophthalmopathy

**DOI:** 10.1038/s41598-020-64402-9

**Published:** 2020-04-30

**Authors:** Sera Park, Dae-Young Park, Jaeryung Kim, Kyung In Woo, Yoon-Duck Kim, Jisang Han, Tae-Young Chung, Hoon-Suk Cha, Dong Hui Lim

**Affiliations:** 1 Samsung Biomedical Research Institute, Seoul, Republic of Korea; 2 Department of Ophthalmology, Samsung Medical Centre, Sungkyunkwan University School of Medicine, Seoul, Republic of Korea; 3 Department of Ophthalmology, Kangbuk Samsung Hospital, Sungkyunkwan University School of Medicine, Seoul, Republic of Korea; 4 Division of Rheumatology, Department of Medicine, Samsung Medical Centre, Sungkyunkwan University School of Medicine, Seoul, Republic of Korea

**Keywords:** Disease model, Eye abnormalities

## Abstract

Inflammation and remodelling of orbital tissue associated with enhanced adipogenesis commonly occur in Graves’ ophthalmopathy (GO), however, the underlying mechanisms that link immune cells and adipocytes in orbital inflammation are not well-known. The primary aim of this study was to elucidate how a genetically determined shift in the T-cell repertoire toward self-reactive T-cells could drive orbital adipogenesis. To induce the T-cell-mediated autoimmune response, SKG mice were intraperitoneally injected with zymosan A once at 8 weeks of age. After three months, orbital magnetic resonance imaging (MRI), histopathologic studies, and *in vitro* analyses were performed to evaluate inflammation and adipogenesis. The eyes of the zymosan A-treated SKG mice displayed proptosis and blepharitis. A detailed analysis of orbital adipose tissue showed enhanced orbital adipogenesis and cellular infiltration compared to controls. In addition, increased secretion of adipokines and other cytokines in the periorbital tissue was observed, together with elevated serum concentration of inflammatory cytokines. Orbital adipogenesis was enhanced in zymosan A-treated SKG mice, a novel mouse model for GO-like inflammatory adipose phenotypes most likely induced by T-cell mediated autoimmune responses. This mouse model gives us the opportunity to examine the underlying molecular mechanisms of enhanced adipogenesis in GO, ultimately providing a potential therapeutic target alternative to conventional GO treatment.

## Introduction

Graves’ ophthalmopathy (GO) is defined as autoimmune inflammatory disorder involving the orbit and periorbital tissues, related to the systemic autoimmune process underlying Graves’ disease^1^. Although GO is generally characterized by periorbital oedema, lid retraction, lid lag, erythema, conjunctivitis, and exophthalmos, it is a potentially vision-threatening eye disease that has perplexed physicians and researchers for a long time^2^. GO usually occurs in hyperthyroidism, however, it sometimes develops in patients under eu- or hypothyroid conditions with chronic autoimmune thyroiditis. The incidence rate of GO is 16 women and 3 men per 100,000 people per year^3^. Approximately ~5% of patients with GO have extreme clinical manifestations with severe pain, inflammation, and vision-threatening compressive optic neuropathy^4^. Immunosuppressive agents, including corticosteroids and orbital radiotherapy, have been the mainstay of treatment in the severe and active phases based on the hypotheses that GO is attributed to the loss of immunological tolerance to the thyroid stimulating hormone receptor (TSHR) and other antigenic proteins and GO results from the linked processes of auto-reactivity and tissue remodeling^5^. Nevertheless, glucocorticoids and orbital radiotherapy only reduce inflammatory signs and symptoms in patients in active phase of GO, minimally affecting proptosis and occasionally causing dose-limiting side effects^6^. This unfulfilled medical need is resulting from the poorly understood underlying mechanisms of the disease; periorbital tissues in GO patients can be obtained only during the chronic phase of the disease because surgical decompression of the orbit is usually performed at that phase.

The SKG mouse line, in which chronic autoimmune responses mediated by CD4^+^ T-cell and spontaneously developed arthritis are induced in a conventional (not clean) environment or by a single zymosan A injection, has been used as an experimental animal model of rheumatoid arthritis and spondyloarthritis^7,8^. The primary cause of the disease is a point mutation in a gene that encodes ZAP-70, a key molecule in signal transduction in T-cells^9^. Alteration of T-cell antigen receptor signal transduction through the aberrant ZAP-70 changes thymic selection thresholds of T-cells, promoting positive selection of autoimmune T-cells that would otherwise be negatively selected^7^. Meanwhile, a previous study using blood samples of patients with Graves’ disease reported that a single nucleotide polymorphism within the lymphoid tyrosine phosphatase (LYP), a potent inhibitor of signal transduction and activation of T-cells, had a highly significant association with Graves’ disease compared to healthy controls^10^. Considering these interesting reports, we decided to evaluate whether zymosan A-treated SKG mice exhibited GO phenotypes, and if so, the precise molecular mechanism underlying the enlargement of orbital fat.

## Results

### Zymosan A treatment in SKG mice leads to blepharitis and proptosis

**Figure 1:**
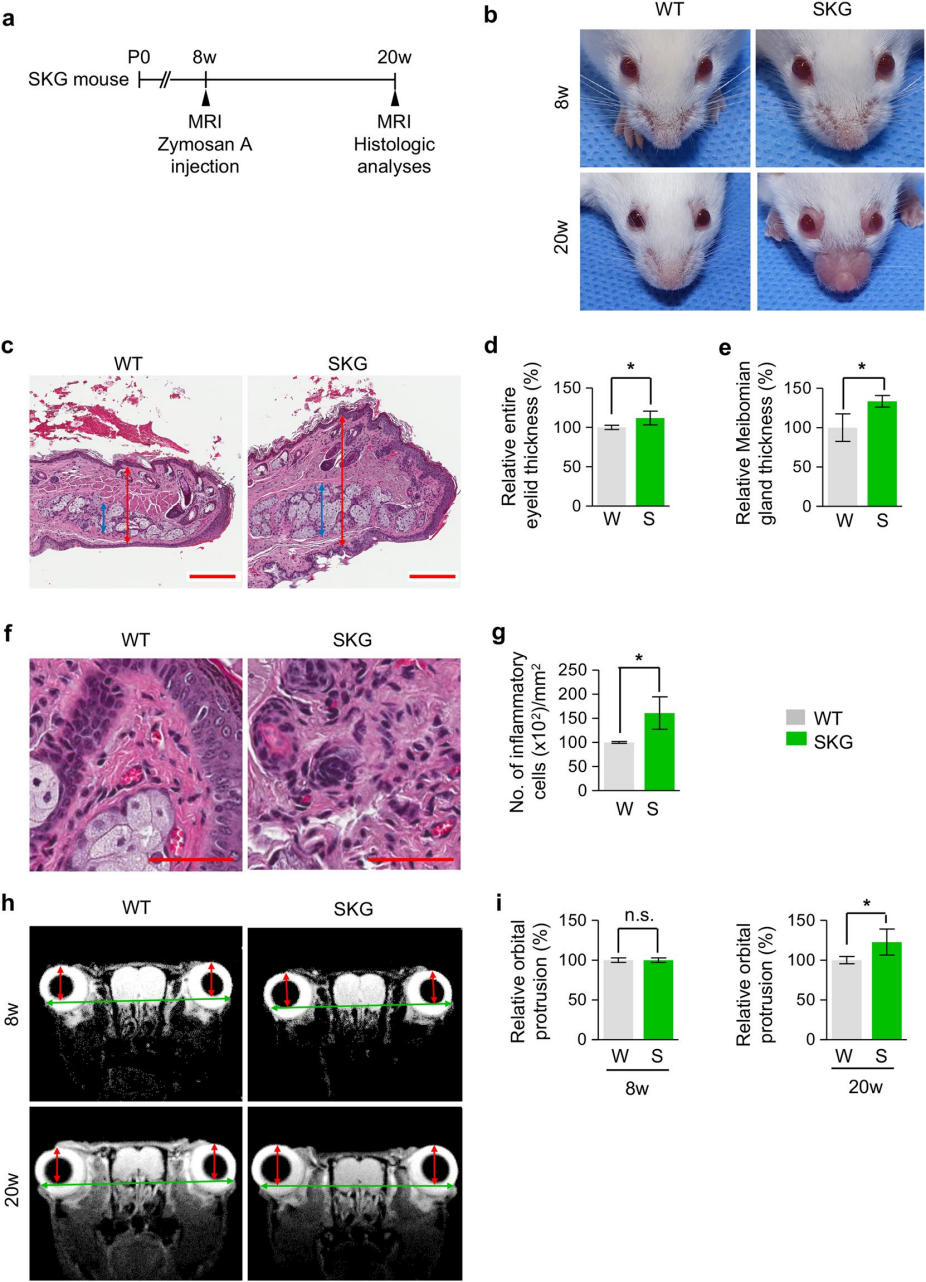
Zymosan A treatment in adult SKG mice encourages blepharitis and proptosis of eyes. (**a**) Diagram of the schedule for an innate immune stimulus by zymosan A administration starting at 8 weeks old and analyses 3 months later using SKG mice. (**b**) Gross images of eyes in SKG and WT mice. (**c**–**e**) Images and comparisons of the thickness of the entire eyelid (red double arrow) and Meibomian gland (blue double arrow). Scale bars: 200 μm. (**f**,**g**) Images and comparison of the number of inflammatory cells in the eyelid. Scale bars: 50 μm. *n* = 3 for each group. *P < 0.05 versus WT by unpaired t - test. (**h**,**i**) MR images and comparison of orbital protrusion measured by the distance (red double arrow) between the corneal apex and the intercanthal line (green double arrow) connecting the bony rims of the outer canthi. *n* = 3 for each group. *P < 0.05 versus WT by unpaired t-test. n.s., not significant.

To investigate whether zymosan A-treated SKG mice induce GO phenotypes, we induced an innate immune stimulus by intraperitoneal administration of zymosan A into 8-week-old SKG mice and analysed their orbits and ocular adnexa 3 months later (Fig. 1a). Normal BALB/c mice among the littermates for each experiment were defined as wild type (WT) mice. The data representative of three independent experiments with three mice in each group were shown unless otherwise indicated. Consistent with a previous finding^11^, we observed a prominent blepharitis phenotype in SKG mice (Fig. 1b). The thickness of the entire eyelid and Meibomian gland were markedly increased by 11.9% and 33.5%, respectively, in SKG mice compared with WT mice (Fig. 1c–e). In addition, increased infiltration of inflammatory cells was observed in the eyelids of SKG mice (Fig. 1f,g), implying that those cells led to blepharitis. Importantly, analysis of images obtained from magnetic resonance imaging (MRI) revealed that eyes of SKG mice displayed significantly greater degree of proptosis by 28.9% in comparison with those of WT mice (Fig. 1h,i). Taken together, these findings indicate that zymosan A treatment in SKG mice induces blepharitis and proptosis, which are similar to GO phenotypes

### Proptosis of eyes in SKG mice is induced by increased inflammation and beige adipogenesis in the orbital fat tissues

**Figure 2:**
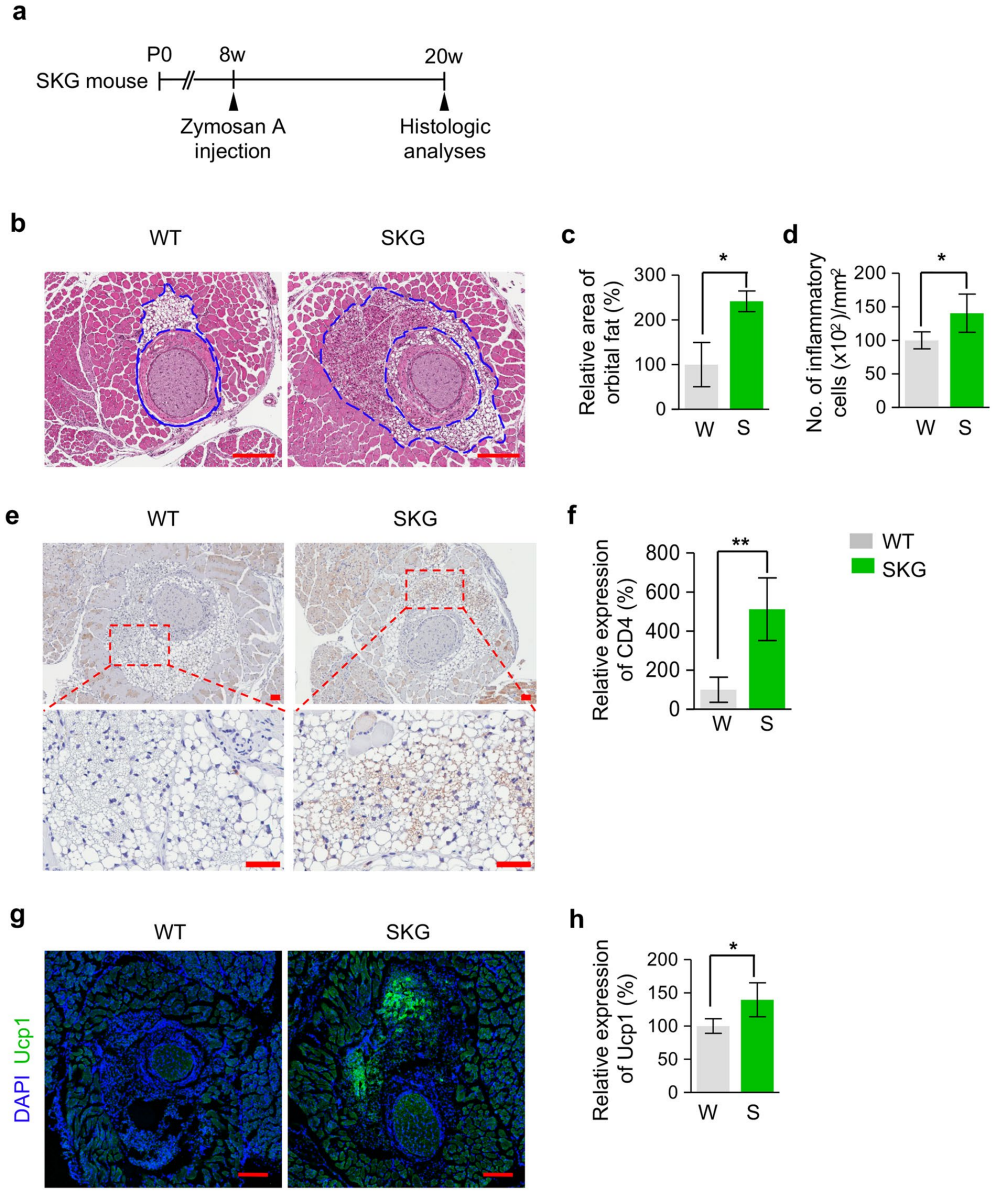
Proptosis of eyes in SKG mice is induced by increased inflammation and beige adipogenesis in the orbital fat tissue. (**a**) Diagram of the schedule for an innate immune stimulus by zymosan A administration starting at 8 weeks old and analyses 3 months later using SKG mice. (**b**–**d**) Images and comparisons of the area of orbital fat around the optic nerve and inflammatory cells of orbital fat around the optic nerve. The blue dashed lines demarcate orbital fat. Scale bars: 200 μm. (**e**,**f**) Images and comparison of the intensity of CD4 immunostaining in periorbital fat. The red dashed boxes are magnified in lower panels. Scale bars: 50 μm. (**g**,**h**). Images and comparison of the intensity of Ucp1 immunostaining in periorbital fat. *n* = 3 for each group. *P < 0.05 and **P < 0.01 versus WT by unpaired t-test.

To elucidate the mechanisms underlying proptosis of eyes in SKG mice, we administered zymosan A into 8-week-old SKG mice and performed histological analyses of orbital tissues 3 months later (Fig. 2a). Intriguingly, compared with WT mice, SKG mice exhibited a markedly increased area of orbital fat around the optic nerve by 140.7% (Fig. 2b,c) while the volume of extraocular muscle and glycosaminoglycan accumulation were not different between zymosan A-treated SKG mice and WT mice (Supplementary Fig. S1), suggesting that proptosis in SKG mice is attributed to enlargement of orbital fat. In addition, increased inflammatory cells in SKG mice were identified only in the periorbital fat (Fig. 2d) except for extraocular muscle (Supplementary Fig. S1). These findings imply that the development of proptosis of the eyes in SKG mice would be ascribed to an inflammatory response in orbital fat. Furthermore, considering that T-cell mediated immune response has a substantial role in adipose tissue metabolism^12,13^ and that self-reactive CD4^+^ T-cells are known to be produced in the thymus of SKG mice^7^, we next investigated the CD4^+^ T-cell distribution in periorbital tissue and the expression of uncoupling protein 1 (Ucp1), a key marker of beige fat^14^, in periorbital fat tissue around the optic nerve. Intriguingly, CD4^+^ T-cells in SKG mice were significantly increased by 412.2% compared with those in WT mice (Fig. 2e,f) and Ucp1^+^ adipocytes in SKG mice were significantly increased by 46.4% compared with those in WT mice (Fig. 2g,h). Together, these findings suggest that increased inflammation and beige adipogenesis by T-cell mediated autoimmune response in the orbital fat tissue of SKG mice could induce proptosis of eyes, resembling GO in humans.

### Increased secretion of adipokines in the periorbital tissue and elevated serum concentration of inflammatory cytokines in SKG mice

**Figure 3:**
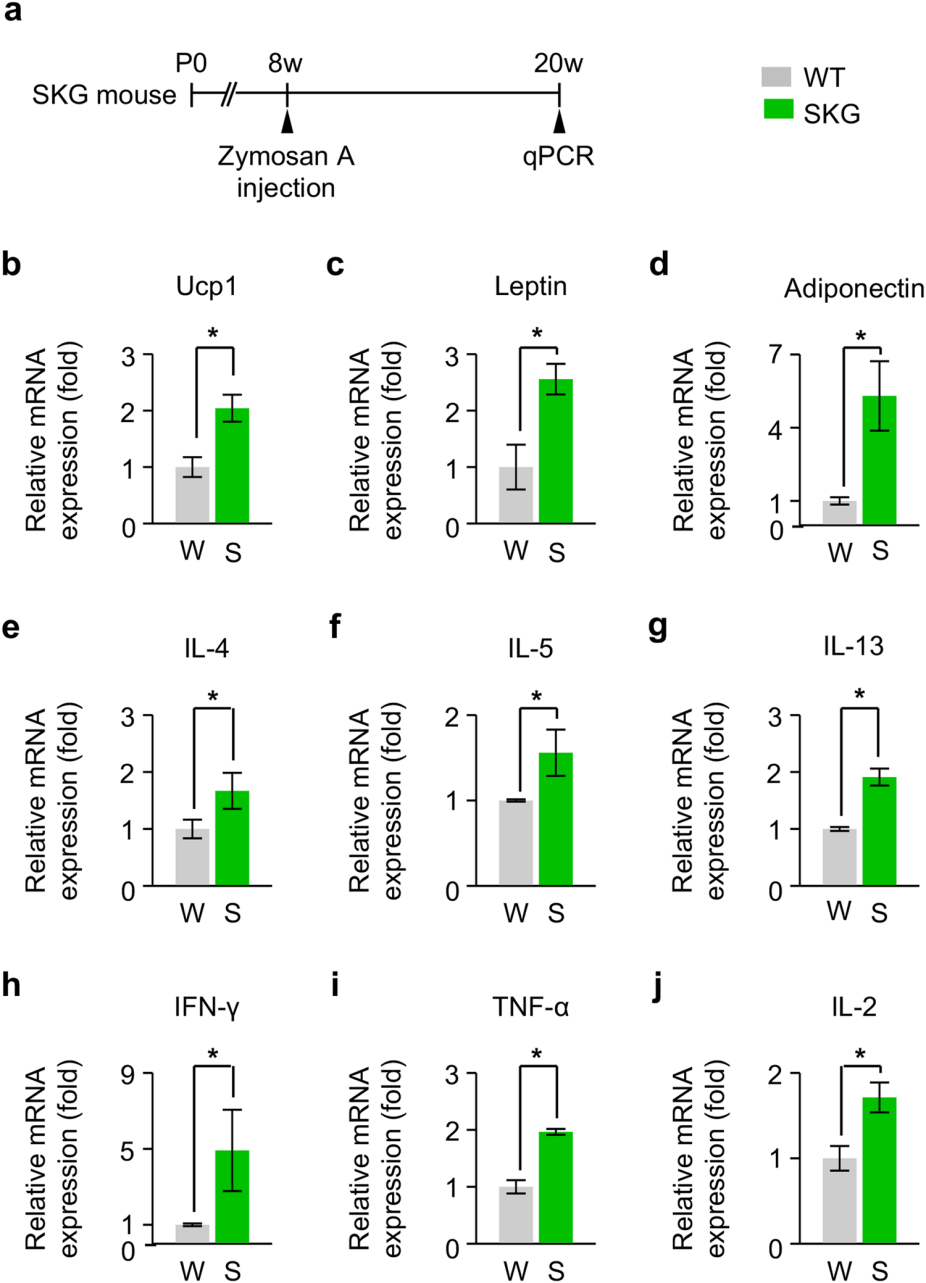
Increased secretion of adipokines in the periorbital tissue of SKG mice. (**a**) Diagram of the schedule for an innate immune stimulus by zymosan A administration starting at 8 weeks old and qPCR analyses of adipokines and other cytokines in the periorbital tissue of SKG mice 3 months later. (**b**–**j**) Relative levels of Ucp1, leptin, adiponectin, IL-4, IL-5, IL-13, IFN-γ, TNF-α, and IL-2. n = 3 for each group. *P < 0.05 versus WT by Mann-Whitney *U* test.

**Figure 4:**
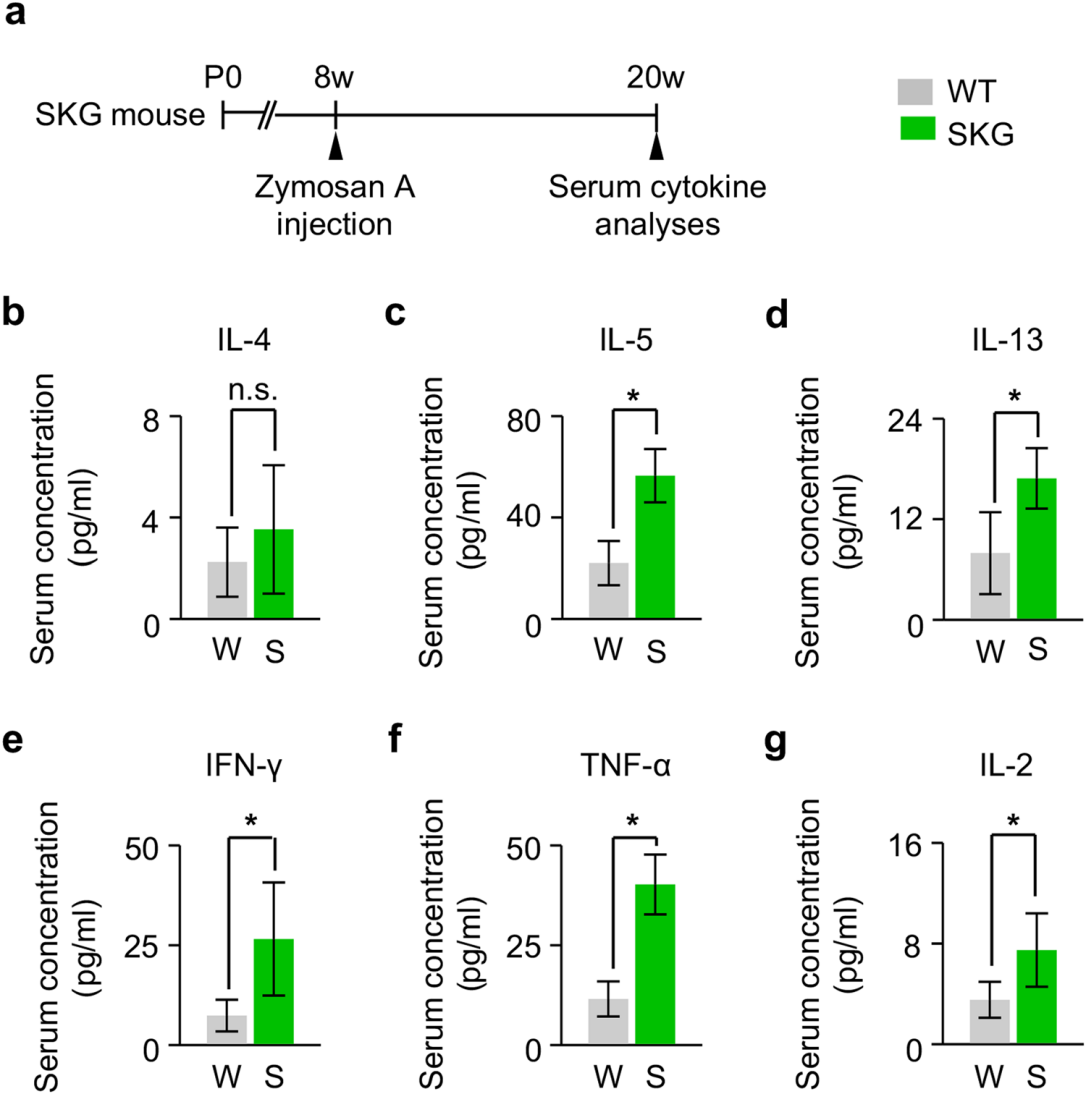
Evaluation of serum concentration of cytokines in SKG mice. (**a**) Diagram of the schedule for an innate immune stimulus by zymosan A administration starting at 8 weeks old and serum cytokine analyses 3 months later using SKG mice. (**b**–**g**) Serum levels of IL-4, IL-5, IL-13, IFN-γ, TNF-α, and IL-2. n = 6 for each group. *P < 0.05 versus WT by Mann-Whitney *U* test.

These findings led us to ask how T-cell mediated autoimmune response in zymosan A-treated SKG mice enhances orbital adipogenesis. To answer this question, we firstly analysed changes in secretion of adipokines in the periorbital tissue of SKG mice at 3 months after zymosan A administration (Fig. 3a). Compared with WT mice, a significantly increased level of several key adipokines, including Ucp1, adiponectin, and leptin, was observed in SKG mice (Fig. 3b–d). In addition, type 2 T-helper (Th2) cytokines, including IL-4, IL-5, and IL-13, were also increased in SKG mice compared with WT mice (Fig. 3e–g), consistent with previous reports demonstrating that beige adipogenesis was induced by Th2 cytokines^15,16^. Meanwhile, periorbital tissues in SKG mice also showed higher expressions of typical pro-inflammatory cytokines, including IFN-γ, TNF-α, and IL-2, compared with those in WT mice (Fig. 3h–j), indicating that pro-inflammatory cytokines also have a certain role in enhanced orbital adipogenesis together with adipokines and Th2 cytokines. We next investigated the serum concentration of cytokines, which were elevated in the periorbital tissue of SKG mice at 3 months after zymosan A injection (Fig. 4a). Most of them were significantly elevated (Fig. 4b–g), implying that both serum Th2 cytokines and pro-inflammatory cytokines would be implicated in the pathogenesis of GO.

## Discussion

In this study, we demonstrate that induction of T-cell-mediated autoimmune response in zymosan A-treated SKG mice results in enhanced orbital adipogenesis. The phenotypes in this mouse model, including proptosis and blepharitis, are involved in GO. The intriguing characteristics of this mouse model are changes of orbital fat and infiltration of inflammatory cells together with increased adipokines and inflammatory cytokines in periorbital tissue and serum. Thus, zymosan A-treated SKG mice could be applied to the study of GO, eventually providing opportunities for searching novel therapeutic targets for GO.

Previously, several murine models of GO have been suggested^17^. Among them, plasmid^18^ or adenovirus-induced^19^ immunization with TSHR in BALB/c mice was known to be the most successfully established murine GO model, which demonstrated inflammatory alterations and increased volumes of orbital fat and muscle. However, considering that autoantibodies to TSHR are not the only causes for GO and the underlying molecular mechanism of GO is extremely complex, we decided to evaluate whether the CD4^+^ T-cell-mediated chronic autoimmune process in the zymosan A-treated SKG mice is involved in the pathogenesis of GO. In this study, compared with a previously established GO mice model, zymosan A-treated SKG mice showed similar phenotypes, including inflammatory responses in orbital tissues and an increase in orbital fat, although no phenotypical difference was found in extraocular muscle. In particular, when we evaluated the concentration of adipokines in the periorbital tissues and serum cytokines in zymosan A-treated SKG mice, elevated levels of both periorbital adipokines and serum cytokines were identified, indicating that this mouse model induces autoimmune-related adipogenesis in periorbital tissue. Therefore, it is supposed that there would be a more suitable, pathogenesis-related murine GO model with more prominent GO phenotypes by combining the TSHR immunization model with zymosan A-treated SKG mice. Further research to evaluate the usefulness of the combined model remains to be done.

Enhanced orbital adipogenesis in GO has been attributed to the intracellular Akt/phosphoinositide 3 kinase signalling mediated by TSHR and insulin-like growth factor-1^20^. Recently, Schluter *et al*.^21,22^ reported that brown fat is widely distributed in the retro-orbital fat tissue of mice, and protein expression of Ucp1 was increased in the orbital adipose tissue of mice immunized with human TSHR. In this study, we also identified that increased protein expression of Ucp1 was identified, solidifying the possibility of browning of adipose tissue as a putative mechanism of enhanced orbital adipogenesis in the pathogenesis of GO.

The SKG mice were previously shown to exhibit periocular manifestations, including blepharitis and conjunctivitis, which is induced by an autoimmune, T-cell-intrinsic response^11^. In this study, we also identified similar periocular phenotypes, such as increased infiltration of inflammatory cells and an increase in the thickness of both the entire eyelid and Meibomian gland, which can be observed in patients with GO^23–25^. Therefore, considering enhanced orbital adipogenesis as well as periocular manifestations, zymosan A-treated SKG mice could be a putative model for GO.

In spite of the strengths above, this study has some limitations including small sample size inherent to the use of littermate controls. In addition, because the titre of autoantibodies and anti-zymosan antibodies was not measured due to no commercially-available ELISA kits for mouse serum, our observations does not completely exclude the possibility of potential involvement of these antibodies in the phenotypes of our mouse model, suggesting that future studies are warranted to assess their involvement.

In conclusion, we demonstrated that zymosan A-treated SKG mice could be a suitable murine GO model because of its autoimmune-related, enhanced orbital adipogenesis. In addition, browning of orbital fat tissue is a putative underlying mechanism of increased periorbital fat tissue. Future studies are warranted to evaluate phenotypes of a combined model induced by immunization of TSHR and injection of zymosan A in the SKG mice.

## Methods

### Mice

SKG mice purchased from CLEA Japan were bred in our specific-pathogen-free animal facility in the Samsung Biomedical Research Institute under climate-controlled conditions with a 12-h light/dark cycle and fed with free access to a standard diet (PMI LabDiet) and water. To induce an innate stimulus in SKG mice, 3 mg of zymosan A (Z4250, Sigma-Aldrich) was intraperitoneally injected once into the 8-week-old SKG mice. All mice were anesthetized by intramuscular injection of 40 mg/kg ketamine and 12 mg/kg xylazine before any procedures. All animal care and experimental procedures were performed in accordance with the guidelines approved by the Institutional Animal Care and Use Committee of Samsung Medical Center (No. 20161121001), and mice were handled according to the Association for Research in Vision and Ophthalmology Statement for the Use of Animals in Ophthalmic and Vision Research and the guidelines and regulations of the Laboratory Animal Research Center at Samsung Medical Center, Sungkyunkwan University School of Medicine.

### Histological analyses

Mice were anesthetized as described above. After perfusion fixation was performed using 4% paraformaldehyde solution, the orbits were removed completely, including adjacent bony structures, to leave their contents untouched. Then orbital tissues were decalcified in EDTA for 24 h. After deparaffinization and dehydration, sections were subjected to staining for haematoxylin and eosin (H&E) and Alcian blue. For the immunohistochemical analysis, sections were treated with 0.5% Triton X-100 solution for 30 min at room temperature. After washing, endogenous peroxidase was quenched with 3% H_2_O_2_ (in methanol) and non-specific binding sites were blocked with 5% normal goat serum in PBS. Then, sections were incubated overnight with anti-CD4 antibody (rabbit origin, 1:200, Abcam, Cambridge, MA, USA), followed by detection with the VECTASTAIN Elite ABS rabbit IgG kit (Vector Laboratories, Burlingame, CA, USA), according to the manufacturer’s protocol, and 3, 3-diaminobenzidine (DAB) was adjusted for colour development. The relative area of orbital fat was calculated as a percentage of adipocyte area divided by its control area using ImageJ software (NIH, Bethesda, MD, USA). The infiltrated Inflammatory cell were visualized in three random fields at 400-fold magnification using an Eclipse E1000 microscope (Nikon, Tokyo, Japan). The cells were counted by using ImagePro-Plus software (Media Cybernetics, Silver Spring, MD, USA).

For immunofluorescent staining of sectioned orbits, the orbits with enucleated eyes were fixed with 4% paraformaldehyde in PBS at room temperature for 6 hours. Fixed orbits were then dehydrated in 20% sucrose solution overnight, embedded in tissue-freezing medium (Leica, Nussloch, Germany), and cut into 10 μm sections. Samples were blocked with 5% donkey serum in PBST (0.3% Triton X-100 in PBS) and then incubated in blocking solution with anti-Ucp1 antibody (rabbit origin, Alpha Diagnostic International, San Antonio, TX, USA) at 4 °C overnight. Following several washes, the samples were incubated at room temperature for 4 hours with Alexa Fluor 488-conjugated donkey anti-rabbit IgG (Abcam, Cambridge, MA, USA). Slides were counterstained with DAPI (1:1000, Abcam, Cambridge, MA, USA) for 5 min. Immunofluorescent imaging procedures were performed using a Zeiss LSM780 confocal microscope equipped with argon and helium-neon lasers (Carl Zeiss, Oberkochen, Germany). Morphometric analysis was performed using ImageJ (NIH). To quantify the relative expression of Ucp1, intensity was measured in adipocyte area. For statistical analysis, the values from three different fields in each orbit were averaged. For comparison of staining intensity, the value was normalized by control and presented as percentage.

### Magnetic resonance imaging (MRI)

*In vivo* MRI was performed on a horizontal bore 7 T MRI scanner (Agilent Technologies Inc., USA). Anaesthesia was induced and maintained in mice by inhalation of a 1-2% isoflurane-oxygen mix throughout imaging. The mouse head was located within a 25 mm internal diameter quadrature MRI volume coil (PulseTeq Ltd, Surrey, UK). T2-weighted MR images were acquired using a fast spin echo (FSE) sequence with repetition time of 4 s; effective echo time (TE) of 60; echo train length of 8, RARE factor of 16; field of view (FOV) of 26 mm ×26 mm; matrix size of 256 × 192 (100 µm in-plane resolution); and 4 averages. Contiguous and coronal 0.61 mm thick slices including the eyes and much of the brain were collected. Twenty-four contiguous 0.4 mm thick, 94 µm in-plane resolution MR images were collected from the surface of the eye towards the back of the eye (perpendicular to the long axis of the eye, similar to the orientation for histological processing), using a FSE sequence with TR of 1400; effective TE of 7.84; FOV of 12 mm ×12 mm; matrix size of 128 × 128 (94 µm in-plane resolution); and 24 averages. Respiration and temperature were monitored throughout MRI, with body temperature maintained at 37 °C using warm air (SA Instruments, Stony Brook, NY, USA). The extent of proptosis of the eyes was evaluated by measuring the distance between the corneal apex and the intercanthal line, which is an axis connecting the bony rims of the outer canthi as previously described^26^.

### RNA extraction and quantitative real-time RT-PCR

Total RNA of periorbital tissues was extracted using TRIzol Reagent (Invitrogen) according to the manufacturer’s instructions. RNA (2 μg) was reverse transcribed into cDNA using a GoScript Reverse Transcription Kit (Promega). Then, quantitative real-time PCR was performed using FastStart SYBR Green Master Mix (Roche) and S1000 Thermocycler (Bio-Rad) with the indicated primers (Supplementary Table 1). The real-time PCR data were analysed with Bio-Rad CFX Manager Software (Bio-Rad) using the delta-delta Ct methods as previously described^27^. GAPDH was used as a reference gene, and the results were presented as relative expression to control

### Assessment of serum cytokine profile

Serum levels of IL-4, IL-5, IL-13, IFN-γ, TNF-α, and IL-2 were assessed by a Luminex assay (ProcartaPlex Mouse plex, eBioscience). Specifically, 50 μL magnetic beads were added in each well of a 96-well plate for 30 seconds, and the liquid was removed. Next, the plate was incubated in serum for 30 minutes and 25 μL of the detection antibody for 30 minutes. Then, 50 μL streptavidin – PE was added and incubated for 30 minutes. Finally, plate analysis was performed on a Luminex^TM^ instrument.

### Statistics

The investigators were blind to the genotypes of animals during experiments. Animals or samples were not randomized during experiments and were not excluded from analyses. All parameters of SKG mice were compared with those of littermate controls. Values were presented as the mean ± standard deviation (SD). Statistical significance was determined by the unpaired t-test or the Mann-Whitney *U* test. Statistical analysis was performed with Graphpad Prism 5 (GraphPad software, Inc) for. windows. Statistical significance was set at *p*-value <0.05.

## Supplementary information

Supplementary information.

## Data Availability

All data generated or analysed during this study are included in this published article (and its Supplementary Information files).
