# Supplementary material for: Enhanced orbital adipogenesis in a mouse model of T-cell-mediated autoimmunity, zymosan A-treated SKG mice: Implications for Graves’ ophthalmopathy
Source: Sci Rep. 2020 Apr 30;10:7329. doi: 10.1038/s41598-020-64402-9 (PMC7193596; doi:10.1038/s41598-020-64402-9)
Supplement: Supplementary file 1 — Supplementary information. [file 41598_2020_64402_MOESM1_ESM.pdf]

Supplementary Information

Enhanced orbital adipogenesis in a mouse model of T-cell-mediated autoimmunity, zymosan A-treated SKG mice: Implications for Graves' ophthalmopathy

Sera Park, Dae-Young Park, Jaeryung Kim, Kyung In Woo, Yoon-Duck Kim, Jisang Han, Tae-Young Chung, Hoon-Suk Cha, Dong Hui Lim

*Corresponding author. E-mail: donghui.lim@samsung.com (D.H.L.) and hoonsuk.cha@samsung.com (H.-S.C.)

This material includes:

Supplementary Fig. S1. SKG mice showed no significant change in extraocular muscle volume, glycosaminoglycan accumulation, and infiltration of inflammatory cells.

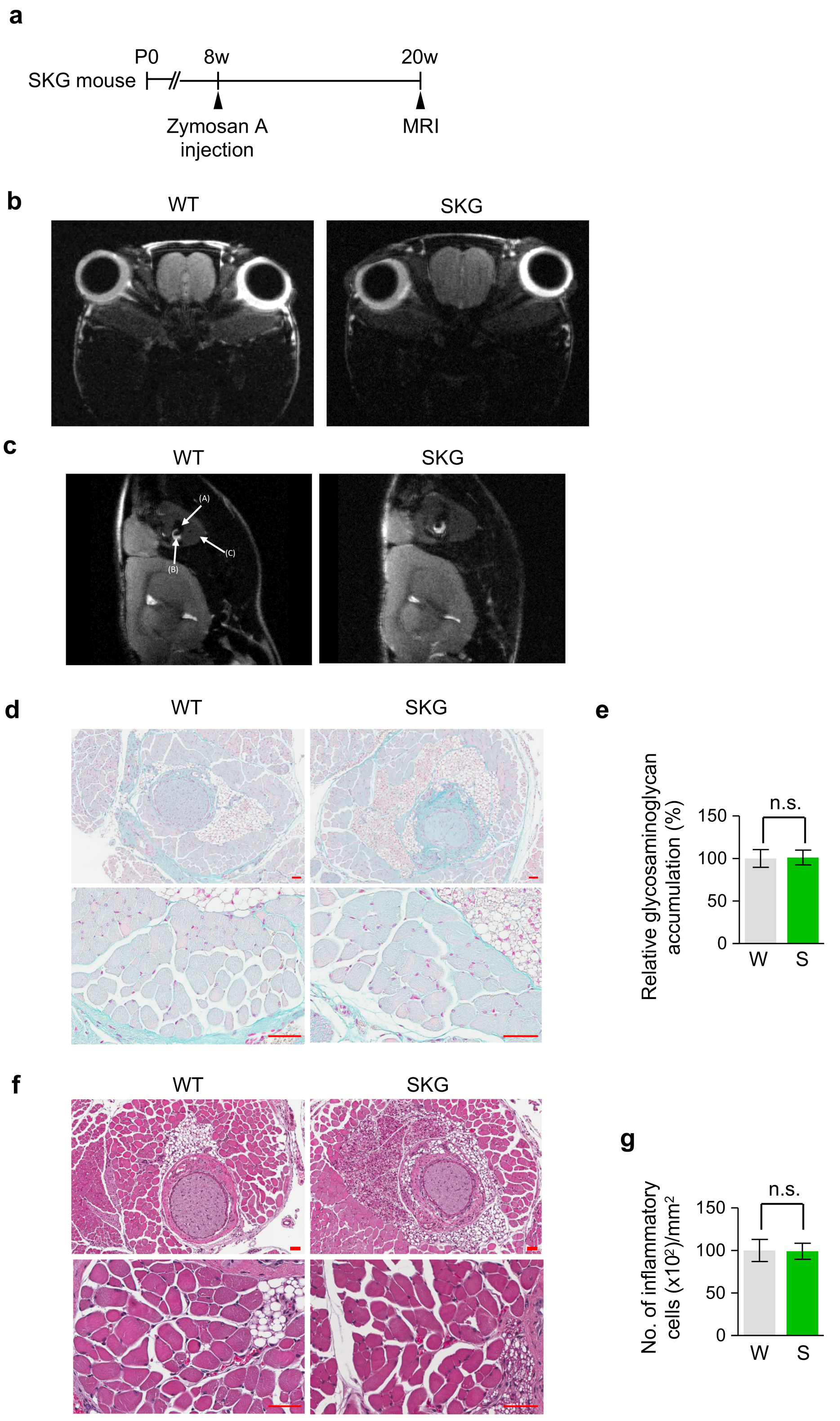

Supplementary Figure 1. SKG mice showed no significant change in extraocular muscle volume, glycosaminoglycan accumulation, and infiltration of inflammatory cells. (a)

Diagram of the schedule for an innate immune stimulus by zymosan A administration starting at 8 weeks old and 3 months later using SKG mice. (**b** and **c**) in vivo MR Images showing the orbital muscles. Extraocular muscle (labelled A), optic nerve (labelled B) and Harderian gland (labelled C). (**d** and **e**) Images and comparison of the intensity of Alcian blue immunostaining for evaluation of glycosaminoglycan accumulation in the extraocular muscles. (**f** and **g**) Images and comparison of the infiltration of inflammatory cells in the extraocular muscles. n = 4 for each group. Scale bars: 50 µm.
